# Healthcare Undergraduates’ Perception and Practice Regarding Lymphatic Filariasis and Mass Drug Administration in a Tertiary Care Facility in Eastern India

**DOI:** 10.7759/cureus.85552

**Published:** 2025-06-08

**Authors:** Bijit Biswas, Hem Nandani Pathak, Pratima Gupta, G. Jahnavi, C. Vasantha Kalyani, Saurabh Varshney

**Affiliations:** 1 Community and Family Medicine, All India Institute of Medical Sciences, Deoghar, IND; 2 Microbiology, All India Institute of Medical Sciences, Deoghar, IND; 3 College of Nursing, All India Institute of Medical Sciences, Deoghar, IND; 4 Otolaryngology, All India Institute of Medical Sciences, Deoghar, IND

**Keywords:** healthcare students, india, lymphatic filariasis, mass drug administration, perception, practice

## Abstract

Background: Undergraduate healthcare students are future frontline communicators in India’s effort to eliminate lymphatic filariasis (LF) through mass drug administration (MDA). This study assessed their perception and practices regarding LF and MDA in an endemic tertiary care setting in Eastern India.

Methods: A cross-sectional survey was conducted in April 2024 among 465 medical and nursing undergraduates using a 39-item self-administered questionnaire. Knowledge scores were calculated for LF (0-20), MDA (0-19), and total (0-39). Data on MDA compliance, drug intake practices, and adverse reactions were also collected.

Results: The mean total knowledge score was 21.2 ± 5.9, with LF and MDA scores of 8.9 ± 3.4 and 12.2 ± 3.7, respectively. Only 37.6% correctly identified 2027 as India’s LF elimination target, and 34.8% were aware of the recommended triple-drug regimen. Although 85.6% reported MDA intake, 25.4% did not follow correct consumption protocols. Among those who took drugs on an empty stomach, 46.9% reported adverse drug reactions versus 26.0% of others (p < 0.001); nausea (39.3%) and headache (29.9%) were most common. Knowledge scores were significantly higher among older students (≥24 years: 24.0 ± 5.5 vs. 18-19 years: 19.5 ± 5.5; p < 0.001), medical stream (21.8 ± 6.0 vs. 18.9 ± 5.2; p < 0.001), and consistent MDA participants (23.7 ± 4.9 vs. 12.5 ± 3.9; p < 0.001).

Conclusion: Despite high MDA coverage, significant gaps exist in knowledge and appropriate drug use. Targeted training and curricular integration are essential to strengthen undergraduate engagement in LF elimination.

## Introduction

Lymphatic filariasis (LF) is a vector-borne neglected tropical disease caused by *Wuchereria bancrofti* and *Brugia malayi* and transmitted primarily by *Culex quinquefasciatus* mosquitoes in India. The disease progresses from asymptomatic infection to chronic manifestations such as lymphoedema, hydrocele, and elephantiasis, leading to lifelong disability, social stigma, and economic hardship [1-3]. Despite substantial global progress, LF remains a major public health concern, with over 657 million people across 39 countries still requiring preventive chemotherapy. India bears the highest burden globally, accounting for nearly 40% of all LF cases [4]. In 2023 alone, the country reported 621,178 individuals living with lymphoedema and 127,100 with hydrocele, with the majority concentrated in high-endemic states including Bihar, Uttar Pradesh, Odisha, Telangana, and Jharkhand. Notably, Jharkhand alone contributed 9% of the national lymphoedema burden and 33% of hydrocele cases, underscoring the urgent need for intensified intervention [2].

To eliminate LF as a public health problem by 2027, three years ahead of the global target, India has adopted an intensified biannual mass drug administration (MDA) strategy, which forms the cornerstone of the national LF elimination program. MDA involves the periodic administration of antifilarial drugs to all eligible individuals in endemic areas, regardless of infection status, aiming to interrupt transmission by reducing microfilarial levels and impairing adult worm reproduction. Two drug regimens are currently in use: the double-drug combination of diethylcarbamazine (DEC) and albendazole (DA), and the triple-drug combination of ivermectin, DEC, and albendazole (IDA), the latter being deployed in selected high-burden districts following WHO endorsement and evidence of superior efficacy [5]. Although administrative coverage has improved over the years, coverage evaluation surveys (CES) continue to reveal significant gaps between drug distribution and actual consumption, often due to fear of adverse drug reactions, mistrust, and inadequate community awareness. Ensuring consumption under direct observation (DOT), strengthening information, education, and communication (IEC) activities, and engaging trusted community figures have been emphasized to address these challenges [6-9].

Healthcare undergraduate students, as future medical and nursing professionals, play a critical role in bridging these gaps. Their responsibilities extend beyond personal compliance with MDA; they are strategically positioned to act as informed advocates and credible sources of health information within their families and communities. However, there is limited empirical evidence regarding their knowledge, perceptions, and behaviors related to LF and MDA [10]. Understanding these dimensions is vital for designing targeted capacity-building and sensitization efforts aimed at leveraging their potential in advancing national LF elimination goals. This study aims to assess the perception and practice of LF and MDA among undergraduate healthcare students in a tertiary healthcare setting in Eastern India, with the broader objective of strengthening their engagement in LF elimination efforts.

## Materials and methods

This cross-sectional observational study was conducted in April 2024 among undergraduate healthcare students at a tertiary care institute in Eastern India. All enrolled undergraduate students from the medical and nursing streams were eligible and invited to participate. A complete enumeration method was employed, including all eligible students present during the data collection period. Assuming that at least 76.1% of the target population would have consumed the annual dose of MDA for LF prevention, based on findings by Biswas et al. [7] from a community-based study conducted in the same geographical area, and considering an absolute precision of 5% and an expected response rate of 75%, the minimum required sample size was calculated to be 374 using Statulator [11], an online sample size calculator. However, a total of 465 healthcare students were ultimately enrolled in the study. Among 412 medical students across the 2020 (n = 62), 2021 (n = 100), 2022 (n = 125), and 2023 (n = 125) batches, 367 students responded, yielding an overall response rate of 89.1%, with response rates of 93.5% in 2020 (n = 58), 84.0% in 2021 (n = 84), 87.2% in 2022 (n = 109), and 92.8% in 2023 (n = 116). Among 155 nursing students from the 2021 (n = 60), 2022 (n = 35), and 2023 (n = 60) batches, 98 participated, yielding an overall response rate of 63.2%, with batch-wise rates of 45.0% in 2021 (n = 27), 82.8% in 2022 (n = 29), and 70.0% in 2023 (n = 42). Data were collected using a structured, self-administered questionnaire via Google Forms. The form link was shared during lecture sessions of the respective batches after briefing the participants about the purpose of the study and ensuring voluntary participation.

The questionnaire began with a brief explanation of the study objectives, followed by an informed consent section embedded within the Google Form. Participants could proceed to the questionnaire only after indicating their consent. Otherwise, the form was terminated. No participant declined consent. The tool captured key sociodemographic variables including age, gender, religion, caste, family income, state or region of residence, academic stream, and batch year. A structured, self-administered questionnaire consisting of 39 items was used to assess knowledge and practices related to LF and MDA. The content covered various domains, such as etiology, mode of transmission, vector species, and behavior, affected organ systems, diagnostic methods used in India, objectives of MDA, including prevention, treatment, and halting disease progression, recommended drug combinations, age-specific dosing, and correct modes of drug administration.

Items were presented in multiple-choice, true/false, and one open-ended format. To minimize response bias and prevent patterned responses, items were arranged in a nonsequential order, and correct responses were not unidirectional. In other words, they did not consistently align with “Yes” or “True.” An “I am not aware” option was included to discourage guessing. Scoring was binary: one point was awarded for each correct response, and zero for incorrect or “I am not aware” responses. The total knowledge score had an attainable range of 0-39, based on 39 items. Domain-specific scores were also computed. LF-related knowledge had an attainable score range of 0-20 (items K1-K3, K7-K15, K17, K33-K39), and MDA-related knowledge had an attainable score range of 0-19 (items K4-K6, K16, K18-K32).

MDA-related practices were also assessed, including consumption status categorized as never taken, taken only in previous years, taken for the first time this year, or taken both previously and this year. Reasons for noncompliance, such as lack of trust in drugs, fear of side effects, or unavailability during the campaign, were recorded using a multiple-response format. Participants were asked how they consumed the MDA drugs, whether diethylcarbamazine was swallowed and albendazole was chewed as per protocol, and whether the drugs were taken after meals or on an empty stomach. Any adverse drug reactions (ADRs) experienced post-consumption, such as nausea, headache, fever, or vomiting, were also recorded using a multiple-response format.

The questionnaire was developed in English and reviewed by two public health experts for content and face validity. In the final sample, the internal consistency of the questionnaire was found to be acceptable, with a Cronbach’s alpha of 0.805 for the total knowledge score, 0.751 for LF-related knowledge, and 0.724 for MDA-related knowledge. Data collection was conducted using a structured Google Form, administered during batch-wise sessions in lecture halls following a clear explanation of the study objectives. The form began with an informed consent section. Only those who consented could proceed further. It was time-restricted to 15 minutes, after which responses were automatically submitted. Participation was entirely voluntary, and strict measures were taken to ensure anonymity and confidentiality throughout the process.

Statistical analysis plan

All data were analysed using JAMOVI software (version 2.3.26, jamovi Project, Sydney, Australia). Descriptive statistics were used to summarize the background characteristics of participants. Categorical variables (e.g., gender, caste, region) were presented as frequencies and percentages along with 95% confidence intervals, while continuous variables (e.g., age, knowledge scores) were expressed as mean ± standard deviation. Independent samples t-tests were used to compare mean knowledge scores between two groups (e.g., gender, stream), and one-way ANOVA with Tukey’s post hoc test was applied for comparisons across more than two groups (e.g., age groups, academic batch, region). The chi-square test of independence was used to assess associations between categorical variables, including MDA compliance status. A p-value of <0.05 was considered statistically significant.

## Results

The mean age of the study participants was 20.7 ± 1.6 years, with an age range of 18 to 25 years. Males slightly outnumbered females, comprising 254 (54.6%) and 211 (45.4%) of the sample, respectively. The majority of participants were Hindu (n = 436, 93.8%) and belonged to the medical stream (n = 367, 78.9%). Regarding social background, one-third of the participants (n = 155, 33.3%) belonged to the Other Backward Class (OBC), while 65 (14.0%) were from the Scheduled Caste (SC), and 29 (6.2%) were from the Scheduled Tribe (ST). In terms of geographic origin, one-fourth of the participants (n = 118, 25.4%) were from Bihar, followed by Uttar Pradesh (n = 54, 11.6%), West Bengal (n = 52, 11.2%), and Rajasthan (n = 52, 11.2%) (Table 1). The knowledge regarding LF and MDA ranged between 13.1% and 94.2% (Table 2).

**Table 1:** Distribution of the study participants as per their background characteristics (n = 465) ^†^ Includes West Bengal (52), Odisha (28), Arunachal Pradesh (2), Assam (1), Manipur (1). ^‡^ Includes Uttarakhand (4), Uttar Pradesh (54), Haryana (12), Delhi (5), Jammu & Kashmir (3), Himachal Pradesh (3), Punjab (2). ^||^ Includes Kerala (14), Andhra Pradesh (7), Telangana (7), Karnataka (3), Tamil Nadu (1). ^¶^ Includes Rajasthan (84), Maharashtra (6), Gujarat (1). ** Includes Madhya Pradesh (14), Chhattisgarh (13). Data are presented as n (%), with 95% CI. OBC: Other Backward Class; SC: Scheduled Caste; ST: Scheduled Tribe; CI: confidence interval; USD: United States dollar.

Variable	n (%)	95% CI
Age in completed years		
18–19	116 (24.9)	21.2–29.1
20–21	206 (44.3)	39.8–48.8
22–23	121 (26.0)	22.2–30.2
≥24	22 (4.7)	3.1–7.1
Gender		
Male	254 (54.6)	50.1–59.1
Female	211 (45.4)	40.9–49.9
Religion		
Hindu	436 (93.8)	91.2–95.6
Muslim	23 (4.9)	3.3–7.3
Christian	5 (1.1)	0.5–2.5
Sikh	1 (0.2)	0.0–1.0
Caste		
OBC	155 (33.3)	29.2–37.7
SC	65 (14.0)	11.1–17.4
ST	29 (6.2)	4.4–8.8
Others	216 (46.5)	41.9–51.0
Family yearly income in USD		
<2326	139 (29.9)	25.9–34.2
2326–5815	136 (29.2)	25.3–33.5
5816–11,629	104 (22.4)	18.8–26.4
≥11,630	86 (18.5)	15.2–22.3
Region		
Bihar or Jharkhand	148 (31.8)	27.8–36.2
Eastern Indian states except Bihar & Jharkhand^†^	86 (18.5)	15.2–22.3
Northern Indian states^‡^	81 (17.4)	14.2–21.1
Southern Indian states^||^	32 (6.9)	4.9–9.5
Western Indian states^¶^	91 (19.6)	16.2–23.4
Central Indian states^**^	27 (5.8)	4.0–8.3
Stream		
Medical	367 (78.9)	74.9–82.4
Nursing	98 (21.1)	17.6–25.0
Batch		
2020	58 (12.5)	9.8–15.8
2021	111 (23.9)	20.2–27.9
2022	138 (29.7)	25.7–33.9
2023	158 (34.0)	29.8–38.4

**Table 2:** Distribution of the study participants as per their knowledge regarding lymphatic filariasis and Mass Drug Administration for its prevention (n = 465) Data are presented as n (%), with 95% CI. LF: lymphatic filariasis; MDA: Mass Drug Administration; DEC: diethyl carbamazine, FTS: filaria test strip; GOI: Government of India; CI: confidence interval.

Item	Variable	Correct Response	n (%)	95% CI
K1	What is the causative agent for LF?	A parasite	423 (91.0)	88.0-93.2
K2	What is the type of vector predominantly responsible for the transmission of LF in India?	Culex mosquito	395 (84.9)	81.4-87.9
K3	What is the target year set for the elimination of LF by the GOI?	2027	175 (37.6)	33.3-42.1
K4	MDA for LF is for prevention?	Yes	438 (94.2)	91.7-95.9
K5	MDA for LF is for treatment?	Yes	212 (45.6)	41.1-50.1
K6	MDA for LF is for the prevention of progression of the disease?	Yes	315 (67.7)	63.4-71.8
K7	Which system is/are affected in LF?	Lymphatic system	452 (97.2)	95.3-98.4
K8	Which of the following is the predominant causative organism for LF (in India)?	Wuchereria bancrofti	396 (85.2)	81.6-88.1
K9	To which phylum does the causative organism of LF belong?	Nematodes	327 (70.3)	66.0-74.3
K10	What are the preferred breeding sites for vector of LF?	Dirty water	220 (47.3)	42.8-51.8
K11	What is the peak biting time of the vector for LF?	Midnight	238 (51.2)	46.6-55.7
K12	Is vector of LF zoophilic?	False	61 (13.1)	10.3-16.5
K13	Is the vector of LF exophilic?	True	78 (16.8)	13.6-20.4
K14	Is the vector of LF exophagic?	False	125 (26.9)	23.0-31.1
K15	Is the vector of LF anthrophilic?	True	185 (39.8)	35.4-44.3
K16	Should MDA for LF be taken preferably after meals?	True	338 (72.7)	68.5-76.5
K17	What is the infective stage of the causative agent of LF?	L3 larvae	260 (55.9)	51.4-60.4
K18	Can only DEC be given as MDA to prevent LF in India?	No	386 (83.0)	79.3-86.1
K19	Can only Albendazole be given as MDA to prevent LF in India?	Yes	66 (14.2)	11.3-17.7
K20	Can DEC+ Albendazole be given as MDA to prevent LF in India?	Yes	306 (65.8)	61.4-69.9
K21	Can DEC+ Albendazole + Ivermectin be given as MDA to prevent LF in India?	Yes	162 (34.8)	30.6-39.3
K22	What is the dose of DEC under MDA for LF for <2 years?	Not indicated	124 (26.7)	22.8-30.9
K23	What is the dose of DEC under MDA for LF for 2-5 years?	100 mg (1 tablet)	144 (31.0)	26.9-35.3
K24	What is the dose of DEC under MDA for LF for 6-14 years?	200 mg (2 tablets)	170 (36.6)	32.3-41.0
K25	What is the dose of DEC under MDA for LF for ≥15 years?	300 mg (3 tablets)	282 (60.6)	56.1-64.9
K26	What is the dose of Albendazole under MDA for LF for <2 years?	200 mg	113 (24.3)	20.6-28.4
K27	What is the dose of Albendazole under MDA for LF for 2-5 years?	400 mg	139 (29.9)	25.9-34.2
K28	What is the dose of Albendazole under MDA for LF for 6-14 years?	400 mg	200 (43.0)	38.6-47.5
K29	What is the dose of Albendazole under MDA for LF for ≥15 years?	400 mg	250 (53.8)	49.2-58.2
K30	The way in which DEC in MDA for LF should be consumed?	Deglutition	331 (71.2)	66.9-75.1
K31	The way in which Albendazole in MDA for LF should be consumed?	Chewing	336 (72.3)	68.0-76.1
K32	The way in which Ivermectin in MDA for LF should be consumed?	Deglutition	204 (43.9)	39.4-48.4
K33	Is blood microscopy one of the diagnostic tests for LF in India?	Yes	387 (83.2)	79.6-86.3
K34	Is urine microscopy one of the diagnostic tests for LF in India?	No	230 (49.5)	44.9-53.9
K35	Is the DEC provocation test one of the diagnostic tests for LF in India?	Yes	288 (61.9)	57.4-66.2
K36	Is Filaria Test Strip (FTS) test one of the diagnostic tests for LF in India?	Yes	324 (69.7)	65.3-73.7
K37	Is quantitative buffy coat examination one of the diagnostic tests for LF in India?	No	179 (38.5)	34.2-42.9
K38	Is microscopy of lymph node aspiration one of the diagnostic tests for LF in India?	Yes	308 (65.8)	61.8-70.4
K39	Is microscopy of hydrocoele fluid one of the diagnostic tests for LF in India?	Yes	273 (58.7)	54.2-63.1

**Figure 1:**
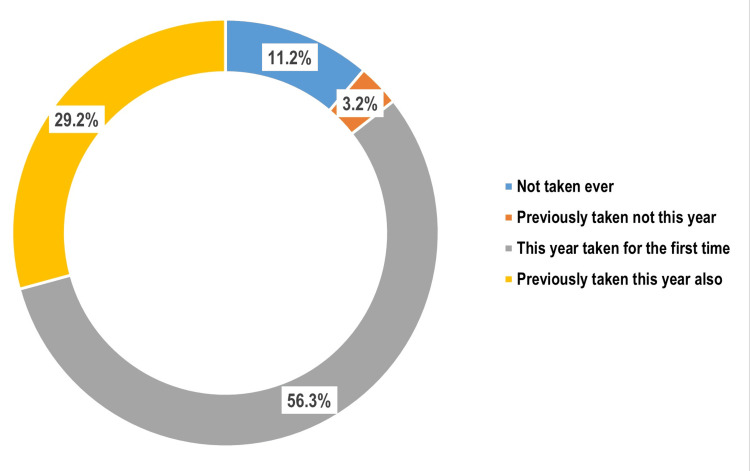
Distribution of the study participants as per their MDA consumption status (n = 465) Data are expressed in percentage (%). MDA: mass drug administration.

**Figure 2:**
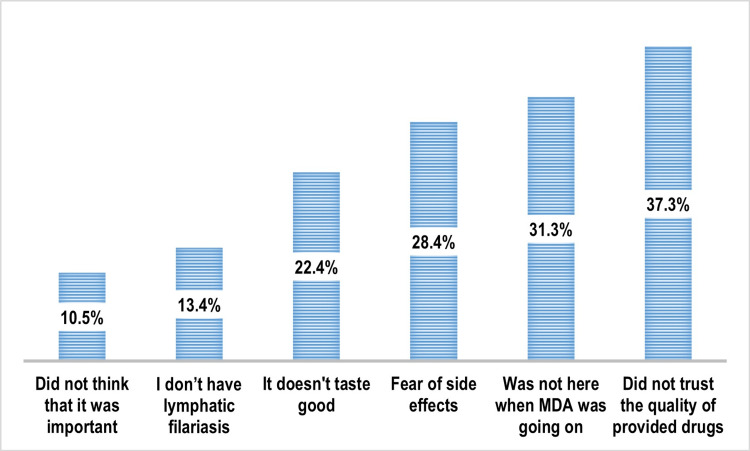
Distribution of the study participants as per the reasons for noncompliance with MDA (multiple response) (n = 67) Data are expressed in percentage (%). MDA: mass drug administration.

Among the study participants, 398 (85.6%) reported having taken MDA, of whom 262 (56.3%) received it for the first time and 136 (29.2%) had taken it in previous years as well. A total of 52 participants (11.2%) had never taken MDA, while 15 (3.2%) had taken it earlier but missed it this year (Figure 1). Among those who did not take MDA in the current year (n = 67), the most commonly cited reason was lack of trust in the quality of the provided drugs (25, 37.3%), followed by being unavailable during the MDA campaign (21, 31.3%), and fear of side effects (19, 28.4%) (Figure 2).

**Figure 3:**
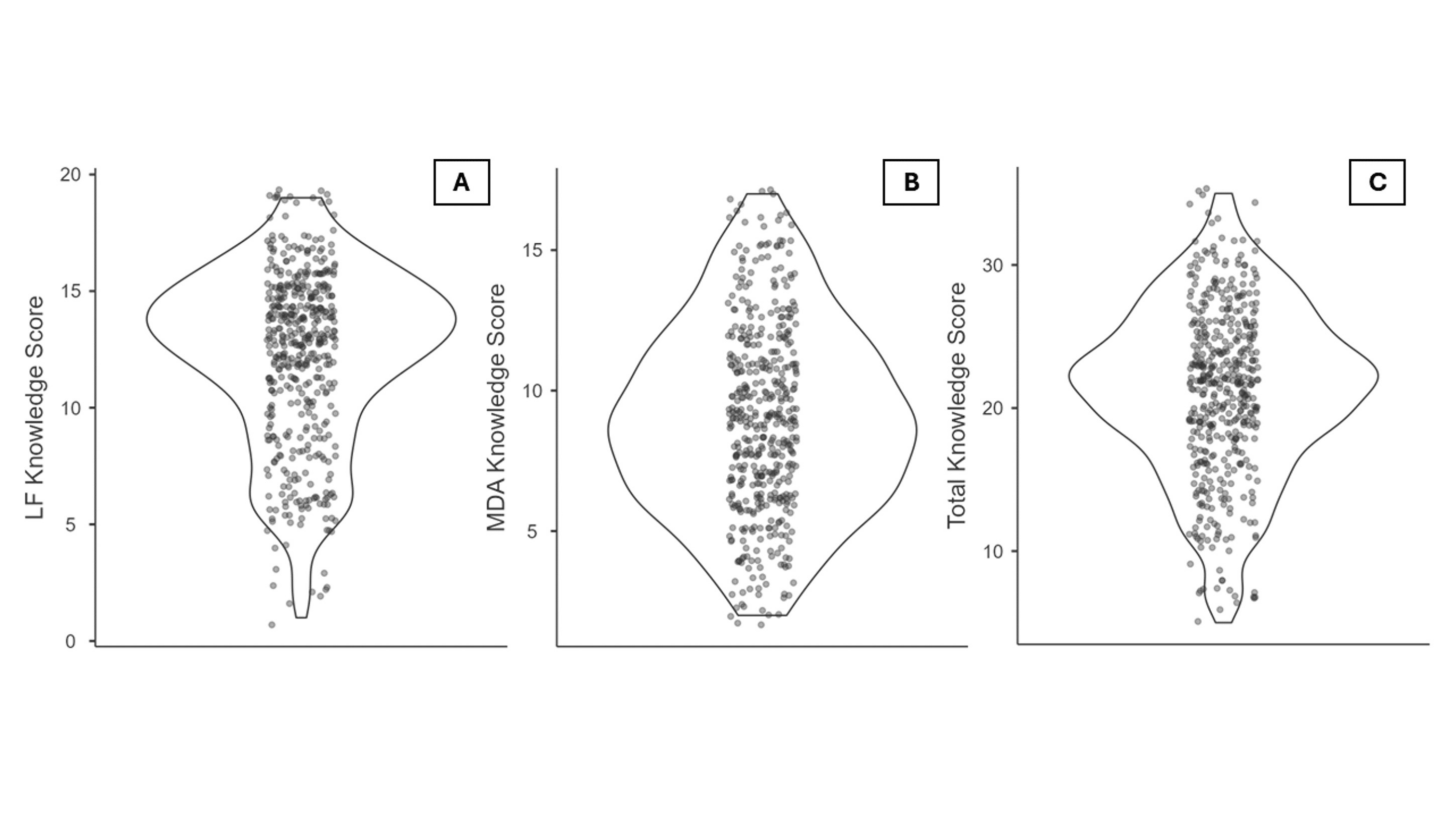
Distribution of the study participants as per their knowledge regarding lymphatic filariasis (LF) and mass drug administration (MDA) for its prevention (n = 465) (A) Knowledge score related to LF. (B) Knowledge score related to MDA for the prevention of LF. (C) Total knowledge score combining LF- and MDA-related knowledge. Data are presented as mean ± SD.

The mean total knowledge score regarding LF and MDA was 21.2 ± 5.9, with scores ranging from 5 to 35. The mean LF-related knowledge score was 8.9 ± 3.4 (range: 2-17), while the mean MDA-related knowledge score was 12.2 ± 3.7 (range: 1-19) (Figure 3). There was a significant association between age and knowledge regarding LF and mass drug administration (MDA). Post hoc analysis revealed that participants aged 22-23 years had significantly higher knowledge of LF compared to those aged 18-19 years (p = 0.047). Similarly, knowledge regarding MDA was significantly higher among participants aged ≥24 years (p = 0.004), 22-23 years (p = 0.001), and 20-21 years (p = 0.016), when compared to the 18-19 age group. When considering the combined knowledge score for LF and MDA, participants aged ≥24 years (p = 0.006) and 22-23 years (p = 0.001) showed significantly greater knowledge than those aged 18-19 years.

A significant association was also found between the region of residence and knowledge regarding MDA. Post hoc comparisons showed that respondents from northern India had significantly higher knowledge of MDA than those from the eastern (p = 0.042) and western (p = 0.014) regions. In terms of overall LF and MDA knowledge, participants from the northern region scored significantly higher than those from the western region (p = 0.047).

Participants from the medical stream demonstrated significantly higher knowledge regarding LF and MDA compared to those from the nursing stream (p < 0.05). Knowledge scores also varied significantly by academic batch. Students from the 2020 batch had significantly higher knowledge of LF compared to the 2022 (p = 0.001) and 2023 (p < 0.001) batches. Similarly, students from the 2021 batch had greater LF knowledge than the 2023 batch (p = 0.002). Regarding MDA knowledge, 2020 batch students outperformed those from 2022 and 2023 (p < 0.001), and 2021 batch students had higher scores than both 2022 (p = 0.014) and 2023 (p < 0.001). Additionally, 2022 batch students had significantly higher MDA knowledge than the 2023 batch (p < 0.001). A similar trend was observed for total knowledge scores related to LF and MDA, with 2020 and 2021 batches performing significantly better than the 2022 and 2023 batches (all p < 0.05).

There was a significant association between MDA compliance and knowledge levels. Participants who had taken MDA for the very first time (p < 0.001) and those who had taken it both in the past and in the current year (p < 0.001) had significantly higher LF knowledge than those who had never taken it. The same two groups also had significantly higher LF knowledge than those who had taken MDA in the past but not during the current year (p < 0.001). Moreover, those who had taken MDA in the past showed higher LF knowledge compared to first-time users (p = 0.008). Similar patterns were noted for MDA knowledge: first-time users (p < 0.001) and those with repeated past and current use (p < 0.001) had significantly higher MDA knowledge. Compared to those who skipped MDA in the current year, first-time users (p = 0.023) and regular users (p = 0.002) had greater MDA knowledge. Overall LF and MDA knowledge was also significantly higher among first-time MDA users (p < 0.001) and regular users (p < 0.001) compared to those who had not taken MDA this year. Lastly, previous MDA users had higher combined knowledge scores compared to first-time users (p = 0.003) (Table 3).

**Table 3:** Distribution of the study participants as per their background characteristics and knowledge regarding lymphatic filariasis and mass drug administration for its prevention (n=465) *ANOVA. ^# ^Independent samples t-test. ^†^ Includes West Bengal (52), Odisha (28), Arunachal Pradesh (2), Assam (1), Manipur (1). ^‡^ Includes Uttarakhand (4), Uttar Pradesh (54), Haryana (12), Delhi (5), Jammu & Kashmir (3), Himachal Pradesh (3), Punjab (2). ^||^ Includes Kerala (14), Andhra Pradesh (7), Telangana (7), Karnataka (3), Tamil Nadu (1). ^¶^ Includes Rajasthan (84), Maharashtra (6), Gujarat (1). ** Includes Madhya Pradesh (14), Chhattisgarh (13). Data are presented as mean ± SD. A p-value of <0.05 was considered statistically significant. OBC: Other Backward Class; SC: Scheduled Caste; ST: Scheduled Tribe; SD: standard deviation; LF: lymphatic filariasis; MDA: mass drug administration; USD: United States dollar.

Variable	Knowledge Score, Mean ± SD								
	Only LF	F/t Value	p-Value	Only MDA	F/t Value	p-Value	Both MDA & LF	F/t Value	p-Value
Age in completed years									
18–19	11.6 ± 3.5	3.209	0.023^*^	7.9 ± 3.3	6.758	<0.001*	19.5 ± 5.5	6.631	<0.001*
20–21	12.0 ± 3.8			9.1 ± 3.5			21.1 ± 6.1		
22–23	12.8 ± 3.8			9.6 ± 3.2			22.4 ± 5.8		
≥24	13.4± 3.3			10.5 ± 3.0			24.0 ± 5.5		
Gender									
Male	12.3 ± 3.7	0.962	0.337^#^	9.2 ± 3.3	1.502	0.134^#^	21.5 ± 5.7	1.463	0.144^#^
Female	12.0 ± 3.8			8.7 ± 3.5			20.7 ± 6.2		
Religion									
Others	11.8 ± 4.4	–0.556	0.578^#^	8.3 ± 2.4	–1.102	0.271^#^	20.1 ± 5.7	–0.979	0.328^#^
Hindu	12.2 ± 3.7			9.0 ± 3.4			21.2 ± 5.9		
Caste									
OBC	12.4 ± 3.3	0.898	0.442*	9.1 ± 3.2	0.136	0.938*	21.5 ± 5.1	0.611	0.608*
SC	11.7 ± 4.2			8.9 ± 3.4			20.6 ± 6.6		
ST	11.5 ± 4.3			8.7 ± 3.8			20.3 ± 7.2		
Others	12.3 ± 3.8			8.9 ± 3.4			21.2 ± 6.1		
Family Yearly Income in USD									
<2326	12.3 ± 3.6	1.157	0.326*	8.8 ± 3.2	0.268	0.849*	21.1 ± 5.6	0.777	0.507*
2326-5815	12.2 ± 3.5			9.0 ± 3.2			21.2 ± 5.4		
5816-11,629	11.7 ± 4.0			8.9 ± 3.6			20.6 ± 6.5		
≥11,630	12.7 ± 3.8			9.2 ± 3.8			21.9 ± 6.5		
Region									
Bihar or Jharkhand	12.2 ± 3.7	0.955	0.445*	9.2 ± 3.3	2.980	0.012*	21.4 ± 5.9	2.109	0.63*
Eastern Indian states except Bihar & Jharkhand^†^	11.8 ± 3.8			8.5 ± 3.4			20.3 ± 5.9		
Northern Indian states^‡^	12.7 ± 4.0			10.0 ± 3.3			22.8 ± 6.2		
Southern Indian states^||^	12.9 ± 3.5			8.2 ± 3.2			21.1 ± 5.1		
Western Indian states^¶^	11.8 ± 3.7			8.4 ± 3.3			20.1 ± 6.1		
Central Indian states^**^	12.5 ± 2.8			9.2 ± 3.8			21.7 ± 5.9		
Stream									
Medical	12.4 ± 3.7	2.406	0.017^#^	9.4 ± 3.5	4.983	<0.001^#^	21.8 ± 6.0	4.362	<0.001^#^
Nursing	11.4 ± 3.7			7.5 ± 2.7			18.9 ± 5.2		
Batch									
2020	14.2 ± 3.7	11.170	<0.001*	11.1 ± 3.2	33.902	<0.001*	25.3 ± 5.8	29.524	<0.001*
2021	12.8 ± 3.8			10.3 ± 3.2			23.1 ± 5.9		
2022	12.0 ± 3.4			9.1 ± 3.2			51.2 ± 5.3		
2023	11.2 ± 3.7			7.2 ± 2.9			18.3 ± 5.2		
MDA status									
Not taken ever	7.1 ± 2.9	67.683	<0.001*	5.3 ± 2.2	32.047	<0.001*	12.5 ± 3.9	79.264	<0.001*
Previously taken, not this year	7.8 ± 3.7			6.9 ± 2.0			14.7 ± 4.2		
Taken this year for the first time	12.7 ± 3.2			9.3 ± 3.1			21.9 ± 4.9		
Previously taken, this year also	13.7 ± 2.8			10.0 ± 3.5			23.7 ± 4.9		

Participants from the medical stream were significantly more likely to be compliant with the annual prophylactic dose of MDA compared to their nursing counterparts (321 (87.5%) vs. 77 (78.6%); p = 0.026). No significant association was observed between MDA compliance and other variables such as age, gender, religion, caste, family income, region, academic stream, or batch (Table 4).

**Table 4:** Distribution of the study participants as per their background characteristics and mass drug administration compliance status (n = 465) *Chi-square test; OBC: Other Backward Class; SC: Scheduled Caste; ST: Scheduled Tribe; SD: standard deviation; LF: lymphatic filariasis; MDA: mass drug administration; USD: United States dollar. ^†^ Includes West Bengal (52), Odisha (28), Arunachal Pradesh (2), Assam (1), Manipur (1). ^‡^ Includes Uttarakhand (4), Uttar Pradesh (54), Haryana (12), Delhi (5), Jammu & Kashmir (3), Himachal Pradesh (3), Punjab (2). ^||^ Includes Kerala (14), Andhra Pradesh (7), Telangana (7), Karnataka (3), Tamil Nadu (1). ^¶^ Includes Rajasthan (84), Maharashtra (6), Gujarat (1). ** Includes Madhya Pradesh (14), Chhattisgarh (13). Data are presented as N, %. A p-value of <0.05 was considered statistically significant.

Variable	MDA Compliance, n (%)		χ² Value	p-Value^*^
	Yes	No		
Age in completed years				
18–19	99 (85.3)	17 (14.7)	0.591	0.898
20–21	175 (85.0)	31 (15.0)		
22–23	104 (86.0)	17 (14.0)		
≥24	20 (90.9)	2 (9.1)		
Gender:				
Male	223 (87.8)	31 (12.2)	2.205	0.138
Female	175 (82.9)	36 (17.1)		
Religion				
Others	24 (82.8)	5 (17.2)	0.201	0.654
Hindu	374 (85.8)	62 (14.2)		
Caste				
OBC	140 (90.3)	15 (9.7)	5.054	0.168
SC	56 (86.2)	9 (13.8)		
ST	23 (79.3)	6 (20.7)		
Others	179 (82.9)	37 (17.1)		
Family yearly income in USD				
<2326	121 (87.1)	18 (12.9)	1.135	0.769
2326–5815	118 (86.8)	18 (13.2)		
5816–11,629	86 (82.7)	18 (17.3)		
≥11,630	73 (84.9)	13 (15.1)		
Region				
Bihar or Jharkhand	130 (87.8)	18 (12.2)	3.308	0.653
Eastern Indian states except Bihar & Jharkhand^†^	70 (81.4)	16 (18.6)		
Northern Indian states^‡^	70 (86.4)	11 (13.6)		
Southern Indian states^||^	27 (84.4)	5 (15.6)		
Western Indian states^¶^	76 (83.5)	15 (16.5)		
Central Indian states^**^	25 (92.6)	2 (7.4)		
Stream				
Medical	321 (87.5)	46 (12.5)	4.962	0.026
Nursing	77 (78.6)	21 (21.4)		
Batch				
2020	54 (93.1)	4 (6.9)	6.834	0.077
2021	98 (88.3)	13 (11.7)		
2022	119 (86.2)	19 (13.8)		
2023	127 (80.4)	31 (19.6)		

**Figure 4:**
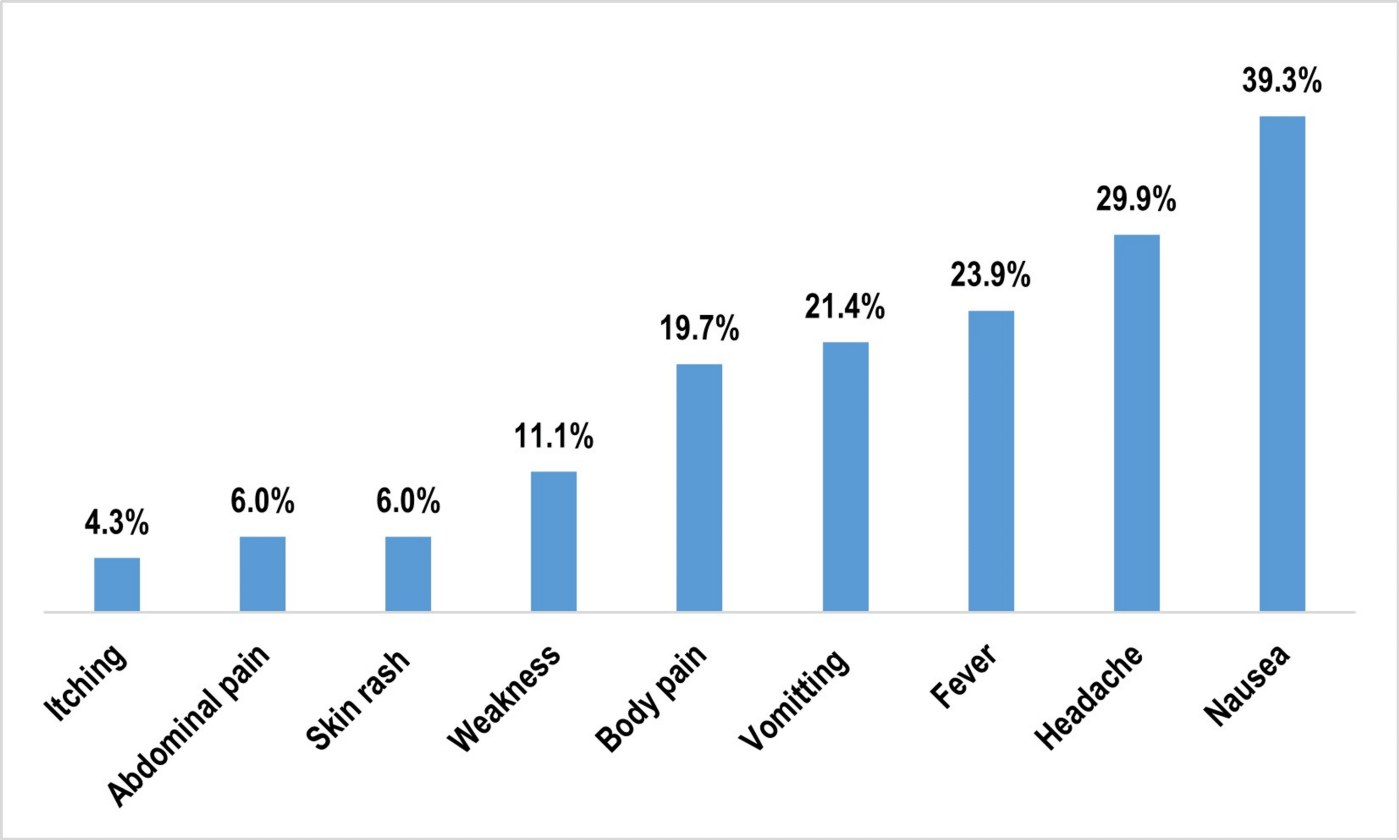
Distribution of the study participants as per the adverse drug reaction post mass drug administration (multiple response) (n = 117) Data are expressed in percentage (%).

Among those who consumed the MDA drugs (N = 398), 333 (83.7%) swallowed diethylcarbamazine (DEC) correctly, while 297 (74.6%) chewed albendazole as instructed. Participants who took the medication on an empty stomach were significantly more likely to experience adverse drug reactions (ADRs) compared to others [30 (46.9%) vs. 87 (26.0%); p < 0.001]. Nausea was the most frequently reported ADR (n = 117), with 46 (39.3%) cases, followed by headache in 35 (29.9%), fever in 28 (23.9%), and vomiting in 25 (21.4%) cases (Figure 4).

## Discussion

This cross-sectional study explored knowledge and practices related to LF and MDA among undergraduate healthcare students in Eastern India. Approximately one in every eight students had either never taken MDA or had missed it during the most recent round. Among those who consumed the drugs, one in four did not follow the correct intake protocol, and nearly one in three who took the drugs on an empty stomach experienced adverse effects, most commonly nausea and headache. Although most students correctly identified LF as a parasitic disease and Culex as the vector, only about one-third were aware of the Government of India’s elimination target year or the correct triple-drug MDA regimen. Students from the medical stream, senior academic batches, and those with consistent MDA participation demonstrated better knowledge scores, indicating that notable gaps persist even among future health professionals.

In the present study, 85.6% of the healthcare students reported consuming MDA drugs. This uptake is comparable to the community-based evaluation by Ratna et al. [12] in Odisha, which reported a similar coverage of 85.8%. However, it was notably higher than the community coverage reported in Deoghar, Jharkhand (76.1%) by Biswas et al. [7], and also exceeded the MDA coverage documented by Mohanty et al. [13] in Odisha (76.2%). Moreover, the uptake in our study was substantially higher than that reported by Bashar et al. [6] in an endemic district of Uttar Pradesh, where only 36.0% of the population consumed the drugs. These comparisons suggest that healthcare students, by virtue of their training and exposure to public health programs, may have better awareness and acceptance of MDA compared to the general community.

In the present study, the leading reasons for noncompliance with MDA were lack of trust in the quality of the provided drugs (37.3%), unavailability during the campaign (31.3%), and fear of side effects (28.4%). Similar findings have been reported in earlier studies: Hussain et al. [14] (77.1%), Mohanty et al. [13] (49.0%), Bashar et al. [6] (34.9%), and Biswas et al. [7] (22.4%), underscoring fear of ADRs as a persistent barrier to MDA uptake. A contributing operational factor may be the mode of albendazole dispensing. In India’s national MDA program, albendazole is supplied in containers of 200 uncoated tablets, while DEC is distributed in blister packs. During field implementation, albendazole is typically dispensed directly into beneficiaries’ hands. This practice is guided by the requirement for DOT, intended to ensure compliance. However, the absence of individual packaging and bare-hand dispensing may raise concerns about hygiene and authenticity among recipients, potentially contributing to distrust in the drug’s quality [15].

Adverse drug reactions were also significantly more frequent among participants who consumed the tablets on an empty stomach (46.9%) compared to those who did not (26.0%). This finding is consistent with Biswas et al. [7], who reported a 64.8% ADR rate among those who took the drugs without food, versus 12.0% with food. This difference is biologically plausible, as DEC is known to cause gastric irritation and nausea when taken without meals. Inadequate counseling on proper intake, including the need to chew albendazole and consume the medication after food, might further exacerbate ADRs and reduce compliance.

In the present study, participants of higher age and senior academic batches demonstrated significantly greater knowledge regarding LF and MDA. Although no directly comparable study among healthcare undergraduates could be found, our findings are consistent with studies in other populations, suggesting a possible influence of maturity, academic progression, and exposure. For example, Joseph et al. [10] in Udupi district, Karnataka, found that healthcare personnel with more years of experience in MDA activities had higher knowledge levels. Likewise, Pramanik et al. [16] reported that secondary and higher secondary school students in Bankura district, West Bengal, showed better awareness of LF, its parasite, and vector compared to primary-level students. However, contrary to our findings, Ifechukwu et al. [17] in Awka, Nigeria, observed no association between age and knowledge of lymphoedema among pharmacy undergraduates, possibly due to contextual or curricular differences.

The study had certain limitations that should be acknowledged. First, it was conducted in a single tertiary healthcare institution in Eastern India, which may limit the generalizability of the findings to other academic or geographic settings. Second, data were collected through a self-administered online questionnaire, which may have introduced recall and social desirability bias, particularly in reporting MDA compliance and adverse drug reactions. Third, although the questionnaire showed acceptable internal consistency, it did not assess underlying attitudes, beliefs, or motivation that may influence MDA uptake and advocacy behaviors. Fourth, the response rate among nursing students was lower compared to medical students, potentially limiting representativeness and affecting comparisons across academic streams. Lastly, the cross-sectional nature of the study restricts causal inferences between knowledge levels and MDA-related practices.

## Conclusions

The study revealed that one in every eight undergraduate healthcare students had never taken MDA or had missed it in the most recent round, despite being part of an endemic region. Among those who consumed the drugs, one in four did not follow correct intake protocols, and nearly one in three who took MDA on an empty stomach reported adverse effects. Although most students recognized LF as a parasitic disease transmitted by Culex mosquitoes, only one in three could identify the target year for elimination or the recommended triple-drug regimen. Knowledge scores were significantly higher among medical students, older participants, senior academic batches, and those who had regularly participated in MDA. These findings highlight persistent gaps in MDA-related knowledge and practices, even among future health professionals, underscoring the need for targeted curriculum integration and IEC strengthening to support India’s LF elimination goals.

## Appendices

**Table 5:** Lymphatic filariasis (LF) and mass drug administration (MDA) knowledge questionnaire

Item	Questions	Responses	Score
K1	What is the causative agent for LF?	I am not aware	0
		A Bacteria	0
		A Virus	0
		A Parasite	1
		A Fungus	0
K2	What is the type of vector is predominantly responsible for transmission of LF in India?	I am not aware	0
		Housefly	0
		Anopheles Mosquito	0
		Hard Tick	0
		Culex Mosquito	1
		Sandfly	0
		Aedes Mosquito	0
		Sof Tick	0
		Manasonia Mosquito	0
		Rat flea	0
		Other vector	0
K3	What is the target year set for elimination of LF by GOI?	________________	1
K4	MDA for LF is for prevention?	I am not aware	0
		Yes	1
		No	0
K5	MDA for LF is for treatment?	I am not aware	0
		Yes	1
		No	0
K6	MDA for LF is for prevention of progression of the disease?	I am not aware	0
		Yes	1
		No	0
K7	Which system is/are affected in LF?	I am not aware	0
		Respiratory system	0
		Cardiovascular system	0
		Lymphatic system	1
		Gastrointestinal system	0
		Other system	0
K8	Which of the following is predominant causative organism for LF (in India)?	I am not aware	0
		Ascaris lumbricoides	0
		Ancylostoma duodenale	0
		Wuchereria bancrofti	1
		Necator americanus	0
		Other causative organism	0
K9	To which phylum the causative organism of LF belongs?	I am not aware	0
		Trematodes	0
		Cestodes	0
		Nematodes	1
		Other phylum	0
K10	What are the preferred breeding sites for vector of LF?	I am not aware	0
		Clean large stream water	0
		Dirty water	1
		Clean small collection of water	0
K11	What is the peak biting time of the vector for LF?	I am not aware	0
		Day	0
		Dawn	0
		Midnight	1
		Dusk	0
K12	Is vector of LF is zoophilic?	I am not aware	0
		True	0
		False	1
K13	Is vector of LF is exophilic?	I am not aware	0
		True	1
		False	0
K14	Is vector of LF is exophagic?	I am not aware	0
		True	0
		False	1
K15	Is vector of LF is anthrophilic?	I am not aware	0
		True	1
		False	0
K16	Is MDA for LF should be preferably taken after meals?	I am not aware	0
		True	1
		False	0
K17	What is infective stage of causative agent of LF?	I am not aware	0
		L1 larvae	0
		L2 larvae	0
		L3 larvae	1
		Stage not specified above	0
K18	Can only DEC be given as MDA to prevent LF in India?	I am not aware	0
		Yes	0
		No	1
K19	Can only Albendazole be given as MDA to prevent LF in India?	I am not aware	0
		Yes	1
		No	0
K20	Can DEC+ Albendazole be given as MDA to prevent LF in India?	I am not aware	0
		Yes	1
		No	0
K21	Can DEC+ Albendazole + Ivermectin be given as MDA to prevent LF in India?	I am not aware	0
		Yes	1
		No	0
K22	What is the dose of DEC under MDA for LF for <2 years?	I am not aware	0
		100 mg (1 tablet)	0
		200 mg (2 tablets)	0
		300 mg (3 tablets)	0
		Not Indicated	1
K23	What is the dose of DEC under MDA for LF for 2-5 years?	I am not aware	0
		100 mg (1 tablet)	1
		200 mg (2 tablets)	0
		300 mg (3 tablets)	0
		Not Indicated	0
K24	What is the dose of DEC under MDA for LF for 6-14 years?	I am not aware	0
		100 mg (1 tablet)	0
		200 mg (2 tablets)	1
		300 mg (3 tablets)	0
		Not Indicated	0
K25	What is the dose of DEC under MDA for LF for ≥15 years?	I am not aware	0
		100 mg (1 tablet)	0
		200 mg (2 tablets)	0
		300 mg (3 tablets)	1
		Not Indicated	0
K26	What is the dose of Albendazole under MDA for LF for <2 years?	I am not aware	0
		100 mg	0
		200 mg	1
		300 mg	0
		400 mg	0
K27	What is the dose of Albendazole under MDA for LF for 2-5 years?	I am not aware	0
		100 mg	0
		200 mg	0
		300 mg	0
		400 mg	1
K28	What is the dose of Albendazole under MDA for LF for 6-14 years?	I am not aware	0
		100 mg	0
		200 mg	0
		300 mg	0
		400 mg	1
K29	What is the dose of Albendazole under MDA for LF for ≥15 years?	I am not aware	0
		100 mg	0
		200 mg	0
		300 mg	0
		400 mg	1
K30	The way in which DEC in MDA for LF should be consumed?	I am not aware	0
		Deglutition	1
		Chewing	0
		Either Deglutition or Chewing	0
K31	The way in which Albendazole in MDA for LF should be consumed?	I am not aware	0
		Deglutition	0
		Chewing	1
		Either Deglutition or Chewing	0
K32	The way in which Ivermectin in MDA for LF should be consumed?	I am not aware	0
		Deglutition	1
		Chewing	0
		Either Deglutition or Chewing	0
K33	Is blood microscopy being one of the diagnostic tests for LF in India?	I am not aware	0
		Yes	1
		No	0
K34	Is urine microscopy being one of the diagnostic tests for LF in India?	I am not aware	0
		Yes	0
		No	1
K35	Is DEC provocation test being one of the diagnostic tests for LF in India?	I am not aware	0
		Yes	1
		No	0
K36	Is Filaria Test Strip (FTS) test being one of the diagnostic tests for LF in India?	I am not aware	0
		Yes	1
		No	0
K37	Is Quantitative buffy coat examination being one of the diagnostic tests for LF in India?	I am not aware	0
		Yes	0
		No	1
K38	Is microscopy of lymph node aspiration being one of the diagnostic tests for LF in India?	I am not aware	0
		Yes	1
		No	0
K39	Is microscopy of hydrocoele fluid being one of the diagnostic tests for LF in India?	I am not aware	0
		Yes	1
		No	0
